# P-1362. Cefiderocol Activity against Carbapenem-resistant *Acinetobacter baumannii-calcoaceticus* complex, Including Molecularly Characterized Clinical Isolates, Causing Infections in United States Hospitals (2020–2023)

**DOI:** 10.1093/ofid/ofae631.1539

**Published:** 2025-01-29

**Authors:** Rodrigo E Mendes, Abigail Scullin, Hank Kimbrough, Maura Karr, Joshua Maher, Cory Hubler, Mariana Castanheira

**Affiliations:** JMI Laboratories, North Liberty, Iowa; Element Materials Technology/Jones Microbiology Institute, North Liberty, Iowa; Element Materials Technology/Jones Microbiology Institute, North Liberty, Iowa; Element Materials Technology/Jones Microbiology Institute, North Liberty, Iowa; Element Materials Technology/Jones Microbiology Institute, North Liberty, Iowa; Element Materials Technology/Jones Microbiology Institute, North Liberty, Iowa; JMI Laboratories, North Liberty, Iowa

## Abstract

**Background:**

Multidrug-resistant (MDR) *Acinetobacter baumannii-calcoaceticus* complex (ACB) have gained attention as an important clinical challenge in the last decades, due to its ability to develop resistance to front-line antibiotics. Cefiderocol (FDC) is a siderophore cephalosporin that uses the iron transport systems of Gram-negative bacteria to optimize cell entry. The activity of FDC and comparators were evaluated against ACB causing infections in US hospitals, including resistant subsets, collected as part of the SENTRY Antimicrobial Surveillance Program.

**Figure:**
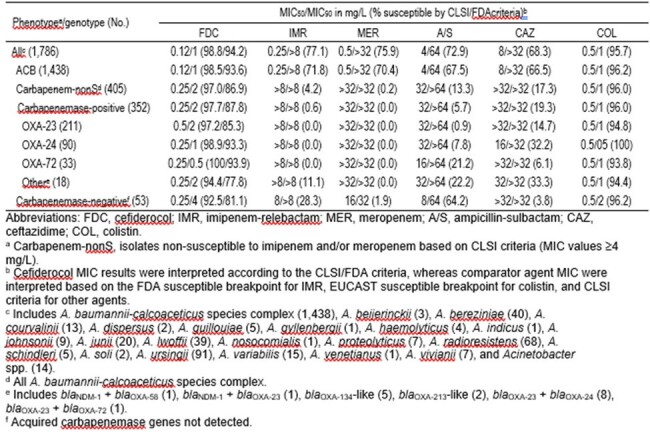

**Methods:**

1,786 *Acinetobacter* spp. were collected from 36 sites in the US during 2020–2023. Susceptibility (S) testing was performed by broth microdilution with cation-adjusted Mueller-Hinton broth (CAMHB) for comparators and iron-depleted CAMHB for FDC. CLSI/EUCAST/FDA criteria were applied. Isolates with imipenem or meropenem MIC ≥4 mg/L (nonS by CLSI) were screened for β-lactamase genes.

**Results:**

ACB comprised 80.5% (1,438/1,786) of isolates collected, and all 405 carbapenem-nonS isolates were identified as ACB. Carbapenemase genes were detected in 86.9% (352/405) of carbapenem-nonS ACB, and *bla*_OXA-23_ (59.9%; 211/352) prevailed, followed by *bla*_OXA-24_ (25.6%; 90/352), and *bla*_OXA-72_ (9.4%; 33/352). FDC (93.6–98.8%S) had MIC_50_ of 0.12 mg/L and MIC_90_ of 1 mg/L against all *Acinetobacter* spp. and the ACB subset (Table). Comparators had limited activity against these isolates (66.5–77.1%S), except for colistin (COL) (95.7–96.2%S). FDC (97.0–97.7%S by CLSI) had MIC_50_ of 0.25 mg/L and MIC_90_ of 2 mg/L against carbapenem-nonS ACB and those carrying carbapenemases. Only COL was also active against these 2 resistant subsets. FDC (97.2–100%S by CLSI) MIC_90_ of 2 mg/L, 1 mg/L and 0.5 mg/L were obtained against ACB carrying *bla*_OXA-23_, *bla*_OXA-24_, and *bla*_OXA-72_, respectively. Isolates carrying other carbapenemases were inhibited by FDC MIC of ≤2 mg/L, except for 1 strain with *bla*_OXA-23_ and *bla*_NDM-1_ (MIC, 8 mg/L).

**Conclusion:**

This study demonstrates the MDR nature of ACB causing infections in US hospitals, and FDC as an active agent against these isolates, regardless of resistance genotype. These *in vitro* data suggest FDC as an important option for the treatment of infections caused by MDR ACB.

**Disclosures:**

**Rodrigo E. Mendes, PhD**, GSK: Grant/Research Support

